# Texas Tech University School of Veterinary Medicine One Health Sciences: Engaging the future^☆^

**DOI:** 10.1016/j.onehlt.2025.101123

**Published:** 2025-07-01

**Authors:** John Gibbons, Ryan Williams, Guy Loneragan

**Affiliations:** Texas Tech University - School of Veterinary Medicine, Amarillo, TX 79106, United States

**Keywords:** One Health Sciences, Veterinary Medicine, Texas Tech University

## 1 Introduction

The One Health Sciences PhD Program (ONHL) housed at the Texas Tech University - School of Veterinary Medicine (TTU-SVM) is the first One Health PhD training Program located at a school of veterinary medicine in the US. Further, ONHL and TTU-SVM are located on the Texas Tech University Health Science Center Campus in Amarillo, TX alongside a clinical component of the TTU College of Medicine, and the TTU Schools of Pharmacy, Nursing, and Public Health. The proximity of these medical affiliations provides opportunities for interdisciplinary and interprofessional research collaborations, scholarly strength, a variety of curriculum options for graduate students, and One Health activities such as Disaster Day, shelter service experiences, and the Amarillo Research Symposium. These partnerships and experiences underscore not only the spirit but the practice of animal, human, and environmental health. In addition to the graduate program, TTU-SVM also delivers Doctor of Veterinary Medicine (DVM) curriculum via a distributed model with 3 years of didactic lecture and laboratory instruction and 1 year of clinical rotations at a variety of clinical sites throughout the state of Texas and eastern New Mexico. The DVM program has a long conceptual history to address the shortage of veterinarians in rural and regional communities throughout the state and beyond, and to support the sustainability of the livestock industries. The DVM program was confirmed by the Texas Higher Education Coordinating Board and received initial funding from the State in 2019. From 2019 until the DVM program began delivering curriculum in the Fall of 2021, the TTU-SVM recruited funding, faculty, the inaugural class of students, and built 2, ≈185,000 square foot buildings and facilities to support bench-top and animal research, teaching laboratories and active learning lecture rooms, and service activities as well as faculty and graduate student offices, conference rooms, surgical, necropsy, and imaging suites, core laboratories, cadaver and tissue storage, and short-term and resident small and large animal facilities. The ONHL program welcomed the inaugural class of 25 graduate students in the Fall of 2022, and received their official coding from the TTU Registrar in early 2023. The ONHL curriculum is designed to be a 72-credit hour deep-learning experience rich in special topics and electives and research opportunities within the ONHL, across the Health Science Center complex, and at the main TTU campus and animal facilities in Lubbock. The crosscutting core competencies of the ONHL program are focused on One Health concepts and applications, public policy, quantitative and qualitative statistics, population health and management, communication and leadership, and inquiry, investigation, and innovation. To facilitate course mapping, the core competencies are based upon the growing body of One Health literature [1,2] and more specifically, One Health experiential learning as it applies to veterinary education [3]. The paring of the DVM and ONHL programs is fundamental in light of the multiple roles that veterinarians play in every spoke of the one health wheel.

## 2 Material and methods

Research and scholarly effort at TTU-ONHL has 5 emphasis areas: 1) One Health Sciences, 2) Disease Ecology, Prevention and Management, 3) Molecular Mechanisms of Disease, 4) Andragogical Scholarship, and 5) Sustainability of Animal Agriculture. In the spirit of One Health, most faculty and graduate students have an interdisciplinary approach to hypothesis driven research and there are several collaborative efforts and centers (e.g. Texas Center for Comparative Cancer Research). Overall scholarly activity and effort based upon faculty interests and research output can be divided into the 5 emphasis areas to generate an approximate percentage contribution (Table 1.); however, there are many faculty that are listed in multiple emphasis areas.

**Table 1:** Approximate One Health scholarly contribution of TTU-SVM faculty to the One Health Sciences Program.⁎

Emphasis Area	Examples of research topics	Percentage#
Disease Ecology, Prevention and Management	Bacterial pathogens, forensic pathology, epidemiology, west TX infectious disease	30 %
Molecular Mechanisms of Disease	Pathogenesis, tumorigenesis, disease detection and surveillance	19 %
Andragogical Scholarship	Novel clinical skills models, emotional intelligence, scholarly output	19 %
One Health Sciences	Interconnection of humans, animals and ecosystem, epidemiology, genetics, antibiotic resistance, microplastics	17 %
Sustainability of Animal Agriculture	Novel drugs, welfare, economics, resource management, animal reproduction	15 %

⁎ https://www.depts.ttu.edu/vetschool/research/research-areas/index.php**.**

# Scholarly effort of emphasis area / total scholarly effort X 100 %.

## 3 Results

The 5 research emphasis areas have more specific examples of research topics (Table 1) which are reflective of faculty expertise and experience, resources, animal availability, and local research assets.

## 4 Discussion

Disease ecology and molecular mechanisms of disease are interconnected, and robust areas of research, and in concert represent almost half of the overall scholarly contribution at TTU-SVM, due to the background of the faculty involved and the obvious link to veterinary medicine and one health. Andragogy, is slightly underrepresented among the ONHL faculty; however, many of the TTU-SVM clinical faculty also contribute to research in these areas albeit veterinary student education is the primary target. Sustainability of animal agriculture has an obvious link to the mission of the TTU-SVM to train veterinarians to service the rural and regional communities in Texas and beyond. Further, sustainability of animal agriculture combined with one health sciences provide perhaps the broadest areas of scholarly contribution and are sensitive to the developing and changing “current events” associated with one health.

### 4.1 Other One Health experiences

There are many types of educational opportunities available at North American One Health Institutions ([4]; student associations and clubs, undergraduate and graduate lectures, undergraduate and graduate courses, certificates, undergraduate majors and minor, Master of Science, PhD), some of which also include One Health research and service experiences (Table 2.). A comprehensive updated list of international training, employment, and funding opportunities can be found (and searched) via the One Health Commission (https://www.onehealthcommission.org/**;** https://www.onehealthcommission.org/en/resources__services/oh_opportunities_bulletin_board/).

**Table 2:** One Health Experiences at private and public institutions in North America.

Region	Number	Training⁎
Northeast US	12	4 research, 2 service
Midwest US	14	1 research, 0 service
Southeast US	16	1 research, 1 service
Southwest US	5	2 research, 2 service
West US	8	3 research, 4 service
Canada	8	4 research, 0 service
Mexico	3	1 research, 0 service

⁎ Number of institutions that have one health research or service experiences. For more information, please contact Dr. Tracey Goldstein (Tracey.Goldstein@colostate.edu).

Further, there are growing One Health related employment opportunities in industry, academia, and government. Academic One Health employment may be regional and sporadic while governmental opportunities seem to be gaining considerable traction. The searchable (.gov) databases for the agencies listed below reflect the funding and employment opportunities associated with One Health.

United States Department of Agriculture.

Center for Disease Control.

Department of Homeland Security.

Department of Health and Human Services.

National Oceanic and Atmospheric Administration.

United States Geological Service.

National Aeronautics and Space Administration.

United States Agency for International Development.

## 5 Conclusions

The TTU-SVM and ONHL relationship is timely, unique, interconnected and symbiotic with a combined focus of educating students in veterinary medicine and one health sciences. Although TTU-SVM has served as the financial engine in the initial phases, ONHL has provided funding and research opportunities for faculty, graduate students and veterinary students via the Veterinary Student Summer Scholars and other programs. Faculty interests and funding streams are diverse but efforts are intertwined and goal oriented. There are certainly many areas of one health that are not services directly by TTU-SVM and ONHL such as globalization, climate change, natural and man-made disasters, etc. Although there are many medical assets available for collaboration, the veterinary component provides the foundation for one health sciences at TTU-SVM and will likely be a cornerstone for future one health initiatives at TTU and beyond.

## CRediT authorship contribution statement

**John Gibbons:** Writing – original draft. **Ryan Williams:** Writing – review & editing. **Guy Lonergan:** Writing – review & editing.

## Declaration of competing interest

None.

## Data Availability

No data was used for the research described in the article.
